# Re-Operative Laparoscopic Colorectal Surgery: A Systematic Review

**DOI:** 10.3390/jcm10071447

**Published:** 2021-04-01

**Authors:** Constantine Halkias, Athanasios Zoikas, Zoe Garoufalia, Michalis K. Konstantinidis, Argyrios Ioannidis, Steven Wexner

**Affiliations:** 1 General Surgery Department, Brighton and Sussex University Hospitals, Brighton BN2 5BE, UK; 2 The 2nd Department of Surgery, Sismanoglio General Hospital of Attica, 15126 Athens, Greece; 8an0s_z@windowslive.com; 3 The 2nd Department of Propaedeutic Surgery, Laiko General Hospital, 11527 Athens, Greece; zoegaroufalia@gmail.com; 4 Department of General, Laparoscopic, Oncologic and Robotic Surgery, Athens Medical Center, 15125 Athens, Greece; mikekonstantinidis@gmail.com (M.K.K.); agis.ioannidis@gmail.com (A.I.); 5 Digestive Disease Center, Cleveland Clinic Florida, Weston, FL 33331, USA; wexners@ccf.org

**Keywords:** re-laparoscopy, re-intervention, colorectal surgery

## Abstract

Introduction: Re-operative laparoscopic colorectal surgery is becoming increasingly common. It can be a challenging procedure, but its benefits can outweigh the associated risks. Methods: A systematic review of the literature reporting re-operative laparoscopic surgery was carried out. Retrospective and prospective cohort studies and case series were included, with case reports being excluded. Results: Seventeen articles dated from 2007 to 2020 were included in the systematic review. In total, 1555 patients were identified. Five hundred and seventy-four of them had a laparoscopic procedure and 981 an open re-operation. One hundred and eighty-three women had a laparoscopic operation. The median age ranged from to 44.9 years to 68.7 years. In seven studies, the indication of the index operation was malignancy, one study regarded re-laparoscopy for excision of lateral pelvic lymph nodes, and one study looked at redo surgery of ileal J pouch anal anastomosis. There were 16 mortalities in the laparoscopic arm (2.78%) and 93 (9.4%) in the open surgery arm. One hundred and thirty-seven morbidities were recorded in the open arm and 102 in the laparoscopic arm. Thirty-nine conversions to open occurred. The median length of stay ranged from 5.8 days to 19 days in laparoscopy and 9.7 to 34 days in the open surgery arm. Conclusions: Re-operative laparoscopic colorectal surgery is safe when performed by experienced hands. The management of complications, recurrence of malignancy, and lateral pelvic floor dissection can be safely performed. The complication rate is low, with conversion to open procedures being relatively uncommon.

## 1. Introduction

Laparoscopic colorectal surgery has been well-established as the preferred approach to the surgical treatment of colorectal cancer, as it carries significant benefits in terms of postoperative morbidity and oncological outcomes. Several randomized controlled trials [1,2,3,4,5] and systematic reviews [6] have proven the merits of laparoscopic colorectal surgery. Laparoscopic re-operative surgery can be challenging, especially in relation to postoperative complications. There is a lack of evidence supporting a laparoscopic approach to re-operation after colorectal surgery, and the systematic reviews available only concern the management of complications [7,8]. The aim of this study is to systematically appraise the relevant literature to identify the efficacy of re-operative colorectal surgery in all relevant occasions, such as the management of complications, re-operation for recurrent malignancy, or other benign conditions.

## 2. Methods

A systematic review of the literature was performed. Studies reporting a laparoscopic approach in re-operative settings that included adult patients were included. Case reports, abstracts, congress proceedings, and non-English language reports were excluded from our review. Studies with fewer than six patients were also excluded. Outcome measures reported were 30-day mortality, length of stay, and conversion to open procedure. Reversal of Hartmann’s procedure was not included in this systematic review.

### 2.1. Literature Search

Two independent researchers (Z.G. and A.Z.) performed a literature search using OvidSP, MEDLINE, EMBASE, Google Scholar, and Cochrane on 1 July 2020. The search terms used were “re operative” OR “re do” OR “re intervention” OR “salvage” AND “laparoscopic” AND “colorectal”. Review articles were hand-searched to identify any remaining studies. The preferred reporting items for systematic review and meta-analyses (PRISMA) guidelines (Figure S1 Supplementary Materials) were followed [9].

### 2.2. Data Collection and Analysis

The same two independent authors screened titles and abstracts produced through our search strategy, and the full texts of relevant articles were obtained. Eligibility was independently assessed by each author. One of the senior authors acted as a mediator whenever there was a disagreement between the two main reviewers in regards to inclusion or exclusion of a paper. The quality of each study was assessed using the Newcastle–Ottawa Scale (NOS).

Data retrieved from each paper included the year and country of publication, type of study, level of evidence [10], type of index surgery, patient age, and gender, as well as the indication for re-operation. Primary outcomes consisted of postoperative morbidity and mortality. Secondary outcomes were the rate of conversion to open and length of stay. Excel^®^ (Microsoft, Redmond, WA, USA) was used for data handling and analysis. Each author was independent and blinded at the time of the data extraction. 

## 3. Results

### 3.1. Search Results and Study Characteristics

Eight hundred and ninety-three (893) potential articles were identified from the search of electronic databases. Thirty (30) full-text articles were assessed for eligibility, and twenty-seven (27) articles were included for full-text reading. Seventeen (17) articles dated from 2007 to 2020 were included in the systematic review [11,12,13,14,15,16,17,18,19,20,21,22,23,24,25,26,27]. Five articles were conducted in South Korea, three in Italy, two in the Netherlands, two in Japan, two in the United States of America, one in Spain, one in Denmark, and one in Argentina. In terms of study design, eight cohort studies, five case control studies, and four case series were included (Table 1). We also used the Oxford level of evidence methodology to evaluate the quality of each study [10]. Seven of the studies were level 2b, five level 3b, and four level 4. The characteristics of the studies are included in Table 1. In total, 1555 patients were identified through this systematic review. Five hundred and seventy-four (574) of them had a laparoscopic operation, and nine hundred and eighty-one (981) had an open re-operation. One hundred and eighty-three female patients had a laparoscopic procedure, with data missing from two studies [13,23]. The median age ranged from to 44.9 years to 68.7 years. In seven studies, the indication of the index operation was malignancy, one study regarded re-laparoscopy for excision of lateral pelvic lymph nodes, and one study looked at redo surgery of ileal J pouch anal anastomosis. 

### 3.2. Primary Outcomes

#### 3.2.1. Postoperative Mortality

The 30-day postoperative mortality date was reported for all 17 studies. In the laparoscopic arm, there were 16 mortalities reported versus 93 in the open. This is translated to a 2.78% mortality rate in the laparoscopic arm and 9.4% in the open arm. Twelve studies reported no mortalities in the laparoscopic arm, and nine studies reported no mortalities in the open arm. One large study [16] with 818 patients reported most of the mortalities (89) in the open arm. This finding may be explained from the fact that the open arm had far more patients compared to the laparoscopic method (659 vs. 159, respectively).

#### 3.2.2. Postoperative Morbidity

The 30-day postoperative morbidity was reported in all but two studies [16,24]. There were 137 total morbidities in the open arm and 102 morbidities in the laparoscopic arm. These pooled results are not directly comparable, as there were significant differences in the sample sizes, and more than a few studies did not have an open arm to report morbidities. The results from each study are presented in Table 2.

### 3.3. Secondary Outcomes

#### 3.3.1. Conversion to Open

Thirty-nine conversions to open surgery were reported in the studies included in this systematic review. Two studies did not report about conversions, and four studies reported zero conversions. In Eriksen et al., eleven of the patients included in the open arm were conversions to open that were not included in the intention to treat analysis (Table 3).

#### 3.3.2. Length of Stay

The length of stay was reported in all studies. The mean was reported in all studies apart from Akiyoshi et al., where the median was reported. The mean hospital stay for laparoscopic surgery ranged from 5.8 days to 19 days, with a range of days of hospital stay between 2 to 128. For open surgery, the mean hospital stay ranged from 9.7 to 34, with a range of days of hospital stay of 6 to 195 days. 

## 4. Discussion

Laparoscopy has been well-established for the treatment of colorectal pathology. A laparoscopic approach for re-operation has been used in several settings in the last twenty years. However, due to various factors, including a steep learning curve in the senior generation of surgeons, it has not been an established practice across the world. 

Our study reviewed the available evidence for laparoscopic re-operative colorectal surgery. There was a low methodological quality of most of the studies (Table 1). There was also lack of randomized controlled trials (most trials were assessed to provide the level of evidence from 4 to 2b using the Oxford levels of evidence system). Despite the above limitations, it became clear from the available evidence and the review of the primary outcomes that re-operative laparoscopic colorectal surgery is safe, with a decreased 30-day mortality, morbidity rate, and rate of conversion to open.

A recent systematic review and meta-analysis by Fransvea et al. [8] showed low morbidity, mortality, and conversion rates in the laparoscopic treatment of complications after colorectal surgery. Our systematic review is the first one to approach redo laparoscopic surgery as an entity itself, and not to focus only on complications. We have included studies also examining lateral pelvic lymph node dissection [17], recurrence [14], rectal surgery as such [25], and redo ileo-anal pouch surgery. In view of the different indications for operations included in each study, our primary and secondary outcomes were chosen with the reproducibility and safety of the intervention in mind.

Redo laparoscopic surgery, regardless of the intervention, has been proven to have a low mortality rate in previous studies [1,5,8]. Our limited pooled data analysis showed a 2.78% mortality rate in the laparoscopic arm compared to 9.4% in the open arm. Vennix et al. [16], who were looking at the management of complications, showed a 4.4% mortality in the laparoscopic arm vs. a 13.6% mortality in the open arm (*p* = 0.001). This was by far the largest and most well-designed cohort study that offers quality data establishing the low mortality rate of laparoscopic management of complications in colorectal surgery. As one could expect, studies that did not investigate immediate complications [14,17,25,26] had zero mortality in the laparoscopic arm. This can be explained from the physiological status of the patients who underwent redo surgery, as it was mainly in an elective or semi-elective setting.

The 30-day morbidity rate was also reported to be lower in the laparoscopic arm. Due to the limitations of our study in not performing a meta-analysis, we are unable to comment on the statistical significance of this pooled difference. Four studies did provide *p*-values of the comparison in the morbidity between the laparoscopic and the open arm. Wind et al. [11] reported four morbidities in the laparoscopic arm and 12 in the open arm, with a *p*-value of 0.087, which was not statistically significant. Roholtz et al. [12] reported a 6% laparoscopic morbidity rate vs. a 30% open morbidity rate (*p* = 0.093); Kwak et al. reported 38.5% vs. 51.5% (*p* = 0.321) [15], and Woo et al. [23] reported 31.6% vs. 42.6% (*p* = 0.403) laparoscopic vs. open morbidity rates, respectively. In all of the above studies, there was no statistically different result in terms of morbidity. Fransvea et al. [8], in their meta-analysis with the limitation of a fixed effect model, did show a statistically significant difference favoring laparoscopy (OR = −0.97; 95% CI −1.63 to −0.29; Z = −2.84; *p* = 0.005). 

Conversion to open surgery was reported from 0% in various studies to 36.8%. Not surprisingly, the biggest conversion ratio (7/19) was in the laparoscopic redo IPAA arm of the Yellinek et al. study [26]. As most studies addressed re-operative laparoscopic surgery on the basis of complications/recurrence, Akiyoshi et al. [17], who looked at laparoscopic lateral pelvic lymph dissection for local recurrence, did not report any conversions to open surgery. Operating surgeon factors, along with experience and other exogenous factors, can affect the decision to convert to open surgery from laparoscopy. Data regarding the index surgery of each case, and whether it was laparoscopic or open, that was converted to open would have been helpful to assess any possible correlations.

Consistent with previous data on laparoscopic surgery, all of the studies included in this systematic review did show a significantly smaller range of mean days of hospital stay for laparoscopy. Fransvea et al. [8], in their meta-analysis on the laparoscopic treatment of complications, also confirmed the above observations (WMD = −0/0 95% CI −1.04 to −0.76, *p* < 0.001) with statistically significant results. This is not a surprise given the experience we have on previous studies suggesting positive postoperative effects of laparoscopic surgery, such as better pulmonary function and reduced postoperative pain.

Our study does carry significant limitations. We decided not to perform a meta-analysis due to the lack of randomized controlled trials. There were significant differences between the sample sizes, surgical expertise, surgical pathology, and indications for operations between studies that also precluded safe conclusions. Due to the lack of subgroup analysis and precise information between open index surgery and re-laparoscopy in most of the studies, we were unable to explore the relationship between open index surgery and the safety of re-laparoscopy. It is important to note that a subset of the studies included in this systematic review concerned specialist surgery (IPAA, laparoscopic lateral pelvic lymph node dissection) that can potentially carry significant morbidity, and is only performed by subspecialists.

## 5. Conclusions

Redo laparoscopic colorectal surgery can be associated with significant benefits, and can be considered equally as safe as open surgery, with potential significant advantages in terms of recovery and mortality. However, these results may be realized only when sufficient laparoscopic expertise exists, and may not be universally applicable. The ability to confer the benefits of laparoscopic re-operative surgery must be decided upon on an individual basis.

## Figures and Tables

**Table 1:** Patient characteristics.

Author, Year [References]	Country	Indication Area	Type of Study	Level of Evidence	Total Patients (Open/Lap)	Female/Male	Age	Index Surgery	Index Surgery Lap/Open	Indication for Re-Operation	NOS
Wind et al., 2007 [11]	Netherlands	Complications	Retrospective study/Case control	3b	10 (25) (15 re-laparotomy vs. 10 re-laparoscopy)	Lap (F:7 M:3) Open (F:8 M:7)	Lap:45 (17–71) Open:45 (20–79)	Mixed (Re-laparo:3malignancy.6 inflammation bowel, 1 diverticulitis)	10 Lap 15 open (open from open)	Anastomotic leak	5
Rotholtz et al., 2009 [12]	Argentina	Complications	Case control	3b	27 (10 re open vs. 17 laparoscopy)	Lap (F:7 M:10) Open (F:6 M:4)	Lap: 61.7+/−18 Open: 57.1+/−16	mixed	Lap	Mixed (12 leak in lap group)	4
Joh et al., 2009 [13]	S. Korea	Complications (leak)	Cohort	2b	19 (17 lap 2 open (previously converted in index operation)	N/A	53.5	Malignancy	Lap	Leak	7
Kwak et al., 2011 [15]	S. Korea	Complications	Case control	3b	57 (31 vs. 26)	Lap (F:3 M:23) Open (F:6 M:25)	Lap 59.0+/− 10.6 open: 61.5+/− 12.3	malignancy	open 1 Lap 23 robot 2	Anastomotic leak	4
Cuccurullo et al., 2014 [19]	Italy	Complications	Retrospective study/Cohort	2b	84 lap all	M 51/F 33 All Lap	64 (32–82) all lap	mixed (including reversal of Hartmann’s)	Lap all	Mixed	6
Lee et al., 2014 [18]	S. Korea	Complications	Retrospective study/Cohort	2b	77 (16 vs. 61)	Open M:14F:2 Lap M:50F:12	Lap 58.5 (37–81) open 60 (49–73)	mixed	Lap all	Anastomotic leak	7
Vennix et al., 2014 [16]	Netherlands	Complications	Retrospective study/Cohort	2b	818 (659 vs. 159)	M/F Lap107 (67.3)/52 (32.7)	Lap 67.0–10.5 open 68.7–11.3	Malignancy (mixed)	Lap all	Mixed	9
Marano et al., 2016 [20]	Italy	Complications	Retrospective study/Cohort	2b	20 lap	M:14F:6 All Lap	67 (47–86) Lap only	Tumor/diverticular disease/pol 17 /1/2	Lap all	Mixed	6
Ibanez et al., 2017 [22]	Spain	Complications	Retrospective study/Case control	3b	40 24 vs. 16)	M:19 Open 9 Lap F: 5 Open 7 LAP	Lap 55.56+/−15.04 [22–80 (Open 67+/−11 44–48)	mal: 14 benign 2	Lap all	Mixed	4
Eriksen et al., 2018 [24]	Denmark	Complications (leak)	Retrospective stud Cohort	2b	87 (51 vs. 36 lap)	Lap (F:14 M:22) Open (F:21 M:30)	Lap 67 (45–88) Open 68 (36–89)	Malignancy	lap all	Anasotmotic leak	8
Numata et al., 2018 [21]	Japan	Complications	Retrospective study/Case Control	3b	31 (16 vs. 15)	Lap M:12F:3 Open M:15F:1	Lap 66 (47–71) Open 68 (55–83)	mal 13LAR 2HAR	lap all	Anastomotic leak	4
Woo et al., 2018 [23]	S. Korea	Complications/REDO ANASTOMOSIS	Prospective study/Case series	4	32 (13 vs. 19)	NO INFO PER ARMtotal M/F 19 (59.3)/13 (40.7)	NO INFO PER ARM overall 60.6 ± 10.6	rectal ca	N/A	Anastomotic leak	Ν/A
Vignali et al., 2020 [27]	Italy	Complications	Retrospective study/ Cohort	2b	lap 23	M/F 3/8	64.1 (13.2) All Lap	mal 16 ben 2	lap right hemi intracorporeal anastomosis	Anastomotic leak	7
Park et al., 2011 [14]	S. KOREA	Recurrence	Retrospective study/Cohort	2b	52 (31 vs. 21)	Lap (F:11 M:10) Open (F:11m:20)	Lap 63 (26–75) Open 58 (29–76)	malignancy	lap 23 open 28	Recurrence & metachronous	4
Gilshtein et al., 2019 [25]	USA	Recurrence/Chronic complications	Retrospective study	2b	78 (56 vs. 22) 4lap/conversion	F/M Lap13/9 Open 22/34	Lap 58.7 (11) Open 59.9 (10)	rectal ca	lap 35 of 78 (45)	Anastomotic leak & recurrence	7
Akiyoshi et al., 2015 [17]	Japan	Local Recurrence (lateral pelvic lymph node dissection)	Case Series	4	9	F:4 M:5 All Lap	56 (48–77)	Malignancy (8 LAR 1 APR)	lap 4 open 5	Isolated local recurrence in the lateral pelvic lymph nodes with likelihood of R0 resection	3
Yellinek et al., 2020 [26]	USA	Redo	Retrospective study	2b	76 (57 vs. lap 19 = 12 + 7 conv)	F/M Lap3/9 Open 29/36	Lap 44.9 (14–72) Open 48.1 (25–79)	IPAA	IPAA	mixed	7

**Table 2:** Primary outcomes.

Author, Year [References]	30-Day Mortality Laparoscopic Arm	30-Day Mortality Open Arm	Morbidity Rate Laparoscopic Arm	Morbidity Rate Open Arm
Wind et al., 2007 [11]	0	0	4 (40%)	12 (80%)
Rotholtz et al., 2009 [12]	0	0	1 (6%)	3 (30%)
Joh et al., 2009 [13]	0	0	2 (11.7%)	1 (50%)
Kwak et al., 2011 [15]	0	1 (3%)	10 (38.5%)	16 (51.6%)
Park et al., 2011 [14]	0	1 (4.7%)	5 (16%)	18 (85.7%)
Cuccurullo et al., 2014 [19]	5	N/A	21 (25%)	N/A
CMLee et al., 2014 [18]	0	1 (6.25%)	26 (42.6%)	16 (100%)
Vennix et al., 2014 [16]	7 (4.4%)	89 (13.5%)	N/A	N/A
Akiyoshi et al., 2015 [17]	0	0	3 (33.3%)	N/A
Marano et al., 2016 [20]	0	N/A	10 (50%)	N/A
Ibanez et al., 2017 [22]	1 (6.25%)	0	14 (87.5%)	2 (8.33%)
Numata et al., 2018 [21]	0	0	4 (26.6)	11 (68.7)
Eriksen et al., 2018 [24]	2 (5.5%)	1 (1.9%)	N/A	N/A
Woo et al., 2018 [23]	0	0	6 (31.6%)	6 (46.2%)
Gilshtein et al., 2019 [25]	0	0	4 (18.1%)	23 (41%)
Vignali et al., 2020 [27]	1 (4,3%)	N/A	7 (38.8%)	N/A
Yellinek et al., 2020 [26]	0	0	2(10.5%)	29 (50.87%)
Total	16	93	119	137

N/A: not available.

**Table 3:** Secondary outcomes.

Author, Year [References]	Conversion to Open	Length of Stay LAP	Length of Stay OP
Wind et al., 2007 [11]	0 (0%)	9 (6–28)	13 (7–38)
Rotholtz et al., 2009 [12]	3 (17.6%)	11.9	18.1 CI–17.75 TO 5.43
Joh et al., 2009 [13]	1 (5.8%)	19 (13–85)	21 ± 21
Kwak et al., 2011 [15]	N/A	18 (10–23)	18 (15–31)
Park et al., 2011 [14] 2011	5 (23.8%)	Recurrence: 10 (5–24) Metachronous: 11 (10–29)	Recurrence: 17.5 (3–63) Metachronous: 19 (10–87)
Cuccurullo et al., 2014 [19]	5 (5.9%)	7.5 (2–37)	N/A
CM Lee et al., 2014 [18]	5 (8.2%)	24.5 (8–128)	12 (6–114)
Vennix et al., 2014 [16]	N/A	17 (11–26)	23 (14–37)
Akiyoshi et al., 2015 [17]	0 (0%)	12 (8–70)	N/A
Marano et al., 2016 [20]	2 (10%)	10 (5–25)	N/A
Ibanez et al., 2017 [22]	2 (12.5%)	15.63+/−12.90 (2–44)	Ν/A
Numata et al., 2018 [21]	0 (0%)	18 (12–47)	31 (17–45)
Eriksen et al., 2018 [24]	0 (0%)	16 (6–57)	34 (4–78)
Woo et al., 2018 [23]	0 (0%)	12 (6–36)	19 (9–195)
Gilshtein et al., 2019 [25]	4 (18.1%)	6.7 (4.2)	9.7 (5.3)
Vignali et al., 2020 [27]	5 (21.4%)	15.5 (9–53)	Ν/A
Yellinek et al., 2020 [26]	7 (36.8%)	5.8 (1.8)	9.7 (3.6)

LAP: laparoscopic procedures, OP: open procedures, N/A: not available.

## Supplementary Materials

The following are available online at https://www.mdpi.com/article/10.3390/jcm10071447/s1, Figure S1: PRISMA Flow Diagram of Study.
