# Supplementary material for: Re-Operative Laparoscopic Colorectal Surgery: A Systematic Review
Source: J Clin Med. 2021 Apr 1;10(7):1447. doi: 10.3390/jcm10071447 (PMC8036625; doi:10.3390/jcm10071447)
Supplement: Supplementary file 1 [file jcm-10-01447-s001.pdf]

Supplementary Material
Figure S1. PRISMA Flow Diagram of Study

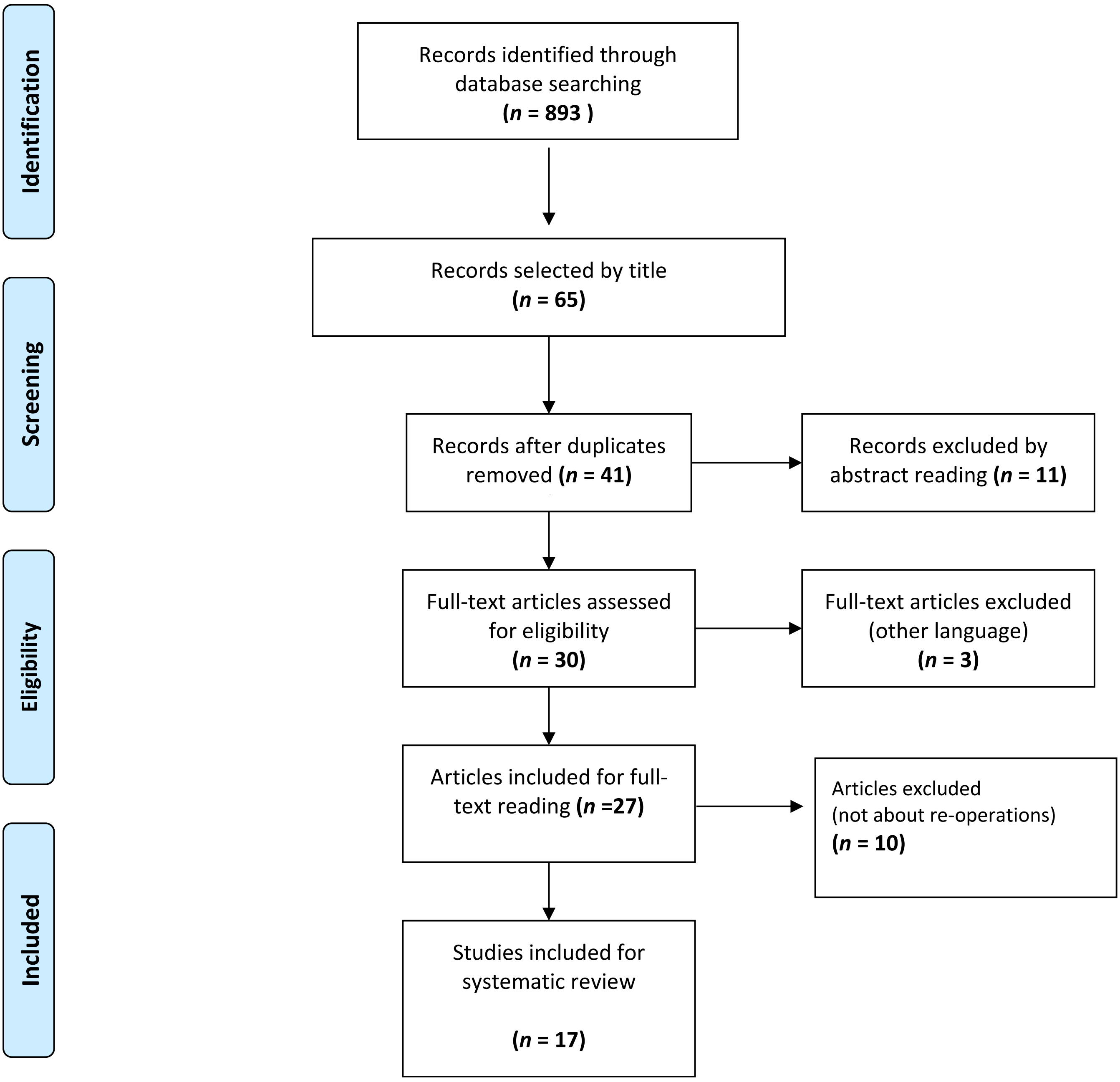

From: Moher, D.; Liberati, A.; Tetzlaff, J.; Altman, D.G.; The PRISMA Group. Preferred Reporting Items for Systematic Reviews and Meta-Analyses: The PRISMA Statement. *PLoS Med.* **2009**, *6*, e1000097, doi:10.1371/journal.pmed1000097.

For more information, visit www.prisma-statement.org.
